# Does obesity or hyperuricemia influence lithogenic risk profile in children with urolithiasis?

**DOI:** 10.1007/s00467-014-2999-9

**Published:** 2014-11-08

**Authors:** Elżbieta Kuroczycka-Saniutycz, Tadeusz Porowski, Piotr T. Protas, Marta Pszczółkowska, Halina Porowska, Jan K. Kirejczyk, Anna Wasilewska

**Affiliations:** 1 Department of Pediatrics and Nephrology, Medical University of Białystok, ul. Waszyngtona 17, 15-274 Białystok, Poland; 2 Department of Medical Chemistry, Medical University of Białystok, Białystok, Poland; 3 Department of Pediatric Surgery, Medical University of Bialystok, ul. Waszyngtona 17, 15-274 Białystok, Poland

**Keywords:** Adolescents, Children, Hyperuricemia, Obesity, Overweight, Urolithiasis

## Abstract

**Background:**

There are indications that obesity and hyperuricemia may influence the formation and composition of urinary stones. The aim of our study was to determine the effect of obesity and hyperuricemia on the urinary lithogenic risk profile in a large cohort of pediatric patients.

**Methods:**

The study population comprised 478 children with urolithiasis and 517 healthy children (reference group). We studied the effects of obesity on the lithogenic profile by dividing the patients with urolithiasis into two groups based on body mass index* Z*-score (patients who were overweight/obese vs. those with normal weight for age) and comparing the two groups. To study the effect of hyperuricemia on the lithogenic profile, we divided the patients with urolithiasis into two groups based on the presence or not of hyperuricemia (110 patients with urolithiasis accompanied by hyperuricemia vs. 368 patients with urolithiasis and normal serum uric acid levels) and compared the groups.

**Results:**

Among the children and adolescents with urolithiasis and hyperuricemia, there was a significantly lower excretion of crystallization inhibitors (citrates, magnesium). We also found significantly negative correlations between serum uric acid levels and the urine citrate/creatinine ratio (citrate/cr.; *r* = −0.30, *p* < 0.01), as well as the magnesium/cr. ratio (Mg/cr.; *r* = −0.33, *p* < 0.01). There was no statistically significant differences in the urinary excretion of oxalates, citrates, calcium, phosphorus, magnesium and uric acid between children with urolithiasis who were either overweight or obese and children with urolithiasis who had a normal body weight.

**Conclusions:**

In our pediatric patient cohort, hyperuricemia was associated with a decrease in the excretion of crystallization inhibitors in the urine, but the clinical relevance of this observation needs to be confirmed in future studies. Obesity and overweight had no direct influence on the lithogenic risk profile in the urinary stone formers in our study, but there was an indication that higher serum uric acid may be associated with impairment in renal function, which in turn could influence the excretion of lithogenic parameters.

## Introduction

The incidence of urolithiasis in children, which is associated with considerable morbidity and recurrence, has increased significantly during the past few decades. The prevalence of pediatric urolithiasis is estimated to be 1–5 % and to account for about 1 in 1,000 pediatric hospital admissions in the USA [1, 2]. Recent studies conducted in major pediatric centers in the USA have observed that the incidence of urolithiasis in children is increasing [3]. The etiology of urolithiasis in children is largely unknown, and this increasing incidence has been attributed to many factors, such as metabolic, anatomical, dietary and environmental risk factors [4–8].

One proposal is that the increase in stone disease is due to an increased consumption of salty fast foods or high-protein diets, an increasingly sedentary lifestyle, the global rise in obesity, global warming and climate changes [9]. Although in pediatric patients, infection is considered the primary cause of urolithiasis [10, 11] the etiologic paradigm for urolithiasis in children has shifted from predominantly infectious to metabolic causes [12–15]. Studies over the past few decades have identified metabolic disorders in 33–95 % of pediatric patients with urolithiasis. Many studies have outlined the systematic detection of metabolic risk factors—such as hypercalciuria, cystinuria and hyperoxaluria—in children with urolithiasis [16–19].

Recent epidemiologic studies have shown an increased prevalence of kidney stones in patients with metabolic syndrome and lifestyle-related diseases, such as obesity [20], type 2 diabetes [21] and hypertension [22, 23]. There is supporting evidence that uric acid may play a pathogenic role in metabolic syndrome, with numerous studies reporting an association between elevated serum uric acid and obesity [24, 25]. Although the question of whether obesity may influence the formation and composition of stones has been studied, far too little attention has been paid to this problem in children and teenagers.

The aims of this study were, first, to investigate the urinary lithogenic risk profile in children and adolescents with urolithiasis and, secondly, to determine whether obesity or hyperuricemia influences the excretion of electrolytes in the urine.

## Material and methods

This observational cross-sectional prospective study was undertaken in the Department of Pediatric Nephrology, University Children’s Hospital in Bialystok between 2002 and 2013. The patient group consisted of 478 children and adolescents, aged 3–18 (median 14.79 years) years with urinary stones of different composition. Most of the patients presented with typical renal colic or stone passage, however about 25 % were asymptomatic or diagnosed with erythrocyturia. In all cases, urolithiasis was confirmed by ultrasonography, and in about 60 % of the cases, also by X-ray examination.

The exclusion criteria included acute infection and inadequate 24 h urine collection as assessed by the level of urine creatinine (cr.) excretion (normal 15–25 mg/kg/24 h). Patients treated with any medications known to affect serum uric acid levels and electrolyte excretions were also excluded.

The reference group comprised 517 healthy children and adolescents, aged 3–18 (median 14.59 years) years, with normal urinary excretion levels of citrate, calcium, oxalate and uric acid and without abnormalities in dipstick urinalysis (Bayer Diagnostic, Bridgend, UK). These children and adolescents were mainly volunteers with a past history of primary nocturnal enuresis (65 %), who were not treated with any drugs at the moment of the study, or of inguinal hernia (25 %) who had been approached to participate while attending the academic center or the children of hospital staff. None of the controls reported a family history of urolithiasis, while their recent screening based on ultrasonography excluded urinary stones.

The group of children and adolescents with urolithiasis was subsequently divided into two subgroups: (1) urolithiasis HU(+) consisting of patients with urolithiasis accompanied by hyperuricemia (serum uric acid levels ≥5.5 mg/dL); (2) urolithiasis HU(−), i.e. patients with urolithiasis and normal serum uric acid levels (<5.5 mg/ dL). In addition, we analyzed the results by dividing patients with urolithiasis in two subgroups according to body mass index (BMI)* Z*-score: (1) subjects with urolithiasis and overweight or obesity ( BMI* Z*-score ≥1) and (2) subjects with urolithiasis and normal body weight (BMI* Z*-score <1).

For all participants, careful clinical histories were taken and physical examinations were performed. Body weight and height were measured using a balance beam scale and pediatric wall-mounted stadiometer, respectively, and BMI was calculated as weight (in kilograms) divided by the square of height (meters squared). Age- and height-specific reference values for BMI and height were generated by the least mean squares (LMS) method [26], which characterizes the distribution of a variable by its median (M), the coefficient of variation (S, i.e., the ratio of the standard deviation and mean), and skewness (L) required to transform the data to normality. Evaluation of these parameters is obtained by a maximum-likelihood curve-fitting algorithm to the original data plotted over the independent variable. The formula for calculating the *Z*-score of BMI or height was: LMS − SDS = {[*Y*M(*t*)]L(*t*) − 1}/[L(*t*) × S(*t*)], where SDS is the standard deviation score,* Y* is the individual observation and L(*t*), M(*t*), and S(*t*) are the specific values of L, M and S interpolated for the child’s age and gender. The LMS values were taken from the OLAF study published by Kulaga et al. [27]. After an overnight fasting, 5 ml of venous, peripheral blood was collected from each patient and used as a reference for routine laboratory testing. Isolated serum aliquots were stored at −80 °C for further analysis. The biochemical work-up included serum creatinine, urea, fasting plasma glucose, lipid profile and, serum uric acid concentration.

Serum creatinine concentration was determined by the Jaffe reaction, and uric acid level was assessed using the colorimetric method, both on a Hitachi apparatus. Serum cholesterol levels were determined by the enzymatic method on the Roche Hitachi 912 chemistry analyzer (Roche Diagnostics, Mannheim, Germany). Serum glucose was measured with the Integra 800 chemistry analyzer (Roche Diagnostics). We calculated fractional excretion of uric acid (FEUA) using the conventional equations: [(urine uric acid × serum cr.)/(urine cr. ×  serum uric acid) × 100]. FEUA was expressed as a percentage. Glomerular filtration rate (GFR) was assessed using the updated Schwartz’s formula (GFR), which is recommended in the pediatric population. The estimated GFR (eGFR) was calculated from the Schwartz formula: eGFR (ml/min/1.73 m^2^) = 0.413 × growth (cm)/ serum  cr. (mg/dL).

All study subjects were evaluated using the same standard protocol which also included the request to continue to follow their customary diet. During the 24-h urine collection, urine was stored in sterile, closed containers at 4 °C without the addition of preservatives, and all measurements were conducted within 4 h after the end of the collection period. Urine citrate concentrations were determined by an enzymatic method using a commercial system (Boehringer Mannheim/R-Biopharm, Darmstadt, Germany) and adjusted for creatinine. Urine calcium (Ca) and creatinine were assessed using the Cobar-Integra 800 analyzer and Roche reagents (Roche Diagnostics). We also analyzed the urine Ca/citrate ratio, which is a age-independent parameter [28]. Urine oxalate levels were examined using standard enzymatic–spectrophotometric methodology (product nr. 591D; Trinity Biotech, Berkeley Heights, NJ). Urinary pH was determined using a microcomputer pH meter (model CP-315 M; Elmetron, Zabrze, Poland). The Bonn Risk Index (BRI) is a measure of the risk for urinary calcium oxalate (CaOx) stone formation and was determined according to the method of Laube [29], using a modified analytical system described elsewhere [30]. In brief, after the urinary Ca^2+^ content was determined, the urine was titrated step-by-step with Ox^2-^ on a computer-operated analytical system. At the moment crystallized particles of CaOx caused a 2 % decrease in light transmission, the computer application automatically stopped the titration process. The BRI value was then calculated as BRI = Ca^2+:^:Ox^2-^. Renal ultrasonography was carried out using a high-resolution ultrasound machine (Toshiba SSH-140A; probe Convex 3.75 MHz; Toshiba, Tokyo, Japan) by a trained person.

The protocol was approved by the Bioethics Committee of The Medical University of Bialystok in accordance with the Declaration of Helsinki. Informed consent was obtained from the parents of all participants and children older than 16 years of age.

## Statistical analysis

Statistical analyses were performed using Statistica®, ver. 10.0 PL (StatSoft, Tulsa, OK). Each of the two groups was compared using the chi-square and Fisher exact tests for categorical variables and the *t* test for continuous variables for normally distributed data and the Mann–Whitney test for the data not normally distributed. The correlations were made with Spearmans’ test. A* p* value of <0.05 was considered to be statistically significant.

## Results

The comparisons of demographic, clinical and metabolic data between the urolithiasis and reference group are summarized in Table 1. The median age, body height, weight and BMI* Z*-score did not differ between groups. Biochemical studies showed that the urolithiasis group had higher cholesterol levels than the reference group. The urine excreted by our patients with urolithiasis also contained lower levels of citrate (*p* < 0.01), significantly higher levels of oxalate and Ca corrected for urinary creatinine and had a significantly higher Ca/citrate ratio and BRI (*p* < 0.01).

**Table 1:** Anthropometric, clinical and metabolic characteristics of the patient group (patients with urolithiasis) and reference group

Demographic, clinical and metabolic variables	Reference group (*n* = 517)	Urolithiasis group (*n* = 478)	*p*
Age (years)	14.59 (10.64–16.60)	14.79 (11.14–16.74)	NS
Gender,* n* (female/male)	298/ 219	273/ 205	NS^a^
BMI (kg/ m^2^)	19.49 (17.01–22.02)	19.36 (16.87–22.04)	NS
BMI* Z*-score	0.08 (−0.56–0.79)	0.03 (−0.65–0.83)	NS
Urine volume (mL/kg/24 h)	19.70 (13.90–27.19)	21.26 (14.81–29.17)	<0.05
Creatinine (mg/dL)	0.595 (0.47–0.71)	0.60 (0.48–0.73)	NS
Uric acid (mg/dL)	4.52 (3.83–5.47)	4.53 (3.89–5.32)	NS
Urea (mg/dL)	25 (21–29)	25 (21–30)	NS
Cholesterol (mg/dL)	151.5 (137–170)	161 (138–186)	<0.01
BRI (L^−1^)	0.30 (0.12–0.80)	0.96 (0.14–3.76)	<0.01
Urine pH	6.36 (6.03–6.70)	6.33 (6.03–6.60)	NS
GFR (ml/min/1.73 m^2^)	125.07 (110.49–143.52)	123.60 (102.21–144.23)	NS
Oxalate/cr. (mmol/g cr.)	0.28 (0.19–0.37)	0.46 (0.23–0.69)	<0.01
Citrate/cr. (mg/g cr.)	585.10 (482.18–810)	461.13 (277.64–702.18)	<0.01
Ca/cr. (mmol/g cr.)	2.87 (2.16–3.65)	3.53 (2.07–5.86)	<0.01
P/cr. (mmol/g cr.)	20.36 (16.73–25.45)	19.61 (15.47–26)	NS
Mg/cr. (mmol/g cr.)	3.47 (2.98–4.11)	3.32 (2.59–4.37)	NS
Uric acid/cr.	0.43 (0.36–0.53)	0.44 (0.36–0.57)	NS
Ca/citrate	0.18 (0.13–0.24)	0.32 (0.17–0.59)	<0.01

Data are presented as the median with the interquartile range [IQR; Q1 (lower quartile) and Q3 (upper quartile)] in parenthesis, unless otherwise indicated

BMI, Body mass index; GFR, glomerular filtration rate; BRI, Bonn Risk Index; cr., creatinine; Mg, magnesium; P, phosphorus; NS, not significant

^a^The chi-square statistic was used

Data on the subgroup analyses are shown in Table 2. . Ninety-seven subjects with urolithiasis (20.29 %) were classified as overweight or obese. Of those, 76 were found to be overweight with a BMI* Z*-score of ≥1 and <2, and 21 were obese with a BMI* Z*-score ≥2. The median serum uric acid concentration in the subgroup of patients with urolithiasis and a BMI* Z*-score of ≥1 was significantly higher than that of the subgroup of patients with urolithiasis and a normal body weight {4.98 [interquartile range (IQR) 4.29–5.84] vs. 4.43 (IQR 3.82–5.21) mg/ dL, respectively; *p* < 0.01}. No statistically significant differences between these subgroups were found for urinary excretion of oxalates, citrates, calcium, phosphorus, magnesium and uric acid.

**Table 2:** Metabolic characteristics of patients with urolithiasis and overweight or obesity (BMI* Z*-score ≥1) and with urolithiasis and a normal body weight (BMI* Z*-score <1)

Demographic and metabolic characteristics	Urolithiasis group with BMI* Z*-score ≥1 (*n* = 97)	Urolithiasis group with BMI* Z*-score <1 (*n* = 381)	*p*
Age (years)	14.27 (11.51–16.43)	14.89 (11.14–16.85)	NS
Gender,* n* (female/male)	53/44	221/160	NS^a^
Urine volume (ml/kg/24 h)	18.07 (12.50–23.15)	22.58 (15.84–30.00)	<0.01
Creatinine (mg/dL)	0.60 (0.49–0.73)	0.60 (0.48–0.73)	NS
Uric acid (mg/dL)	4.98 (4.29–5.84)	4.43 (3.82–5.21)	<0.01
Cholesterol (mg/dL)	171 (151–187)	154.5 (135–185)	NS
Glucose (mg/dL)	90 (85–93)	88 (83–93)	NS
BRI (L^−1^)	0.66 (0.14–3.01)	1.13 (0.15–3.87)	NS
Urine pH	6.40 (6.00–6.61)	6.33 (6.05–6.60)	NS
GFR (ml/min/1.73 m^2^)	126.46 (104.41–145.78)	122.66 (100.64–143.67)	NS
Oxalate/cr. (mmol/g cr.)	0.38 (0.24–0.65)	0.47 (0.23–0.71)	NS
Citrate/cr. (mg/g cr.)	461.86 (267.16–713.51)	455.99 (278.10–697.4)	NS
Ca/cr. (mmol/g cr.)	2.77 (1.84–5.48)	3.63 (2.12–5.89)	NS
P/cr. (mmol/g cr.)	19.63 (15.27–25.10)	19.61 (15.54–26.02)	NS
Mg/cr. (mmol/g cr.)	3.13 (2.43–4.04)	3.39 (2.62–4.53)	NS
Uric acid/cr.	0.44 (0.35–0.56)	0.44 (0.36–0.57)	NS
Ca/citrate	0.28 (0.15–0.56)	0.32 (0.17–0.59)	NS

Data are presented as the median with the IQR in parenthesis, unless otherwise indicated

^a^The chi-square statistic was used

The correlation analyses of BMI* Z*-score with clinical parameters (Table 3) revealed that the BMI* Z*-score was positively correlated with serum uric acid (*r* = 0.14, *p* < 0.01) and creatinine (*r* = 0.12, *p* < 0.01) and negatively correlated with eGFR (*r* = −0.13, *p* < 0.01). However, there were no significant correlations between the BMI* Z*-score and urinary lithogenic risk profile. We also found that the BMI was negatively correlated with urine pH (*r* = −0.1, *p* < 0.01), but we didn’t find statistically significant correlations between BMI Z-score and urine pH.

**Table 3:** Correlations between BMI* Z*-score and serum uric acid level and examined parameters

Metabolic parameters	BMI* Z*-score		Uric acid (mg/dL)	
	*r*	*p*	*r*	*p*
Uric acid (mg/dL)	0.14	<0.01*	–	–
Creatinine (g/24 h)	0.12	<0.01*	0.50	<0.01
Urine pH	−0.001	NS	−0.10	<0.01
eGFR (mL/min/1.73 m^2^)	−0.13	<0.01*	−0.16	<0.01
Citrate/cr. (mg/g cr.)	−0.002	0.94	−0.30	<0.01
Mg/cr. (mmol/g cr.)	−0.04	0.23	−0.33	<0.01
Oxalate/cr. (mmol/g cr.)	−0.02	0.52	−0.16	<0.01
Ca/cr. (mmol/g cr.)	−0.03	0.31	−0.13	<0.01
P/cr. (mmol/g cr.)	−0.04	0.20	−0.31	<0.01
Uric acid/cr.	−0.02	0.61	−0.37	<0.01
Ca/citrate	−0.00	0.99	0.13	<0.05

eGFR, Estimated glomerular filtration rate; Ca, calcium

Those children and adolescents with urolithiasis and hyperuricemia had a significantly lower excretion of most electrolytes corrected for urinary creatinine, especially crystallization inhibitors (citrates, magnesium) (Table 4 ). The FEUA has been observed to be lower in patients with hyperuricemia than in those with normouricemia, supporting the notion that the predominant mechanism for hyperuricemia is renal underexcretion of uric acid. Therefore, we also analyzed the relationship between serum uric acid levels and urinary crystallization rates and found significantly negative correlations between serum uric acid levels and the citrate/cr. (*r* = −0.30, *p* < 0.01) and Mg/cr. (*r* = −0.33, *p* < 0.01) ratios. In reference patients with elevated uric acid we also found a lower urine excretion of citrate and magnesium. These results are summarized in Table 5. Similarly, significant differences were found in the urinary excretion of oxalates, citrates, calcium, phosphorus, magnesium and uric acid between the reference patients with hyperuricemia and normouricemia (*p* < 0.01).

**Table 4:** Metabolic characteristics of patients with urolithiasis and hyperuricemia and patients with urolithiasis and normouricemia

Demographic and metabolic parameters	Urolithiasis group (HU+) (*n* = 110)	Urolithiasis group (HU−) (*n* = 368)	*p*
Age,* n* (years)	16.29 (14.93–17.35)	14.24 (9.95–16.48)	<0.01
Gender,* n* (female/male)	34/76	239/129	<0.05^a^
BMI* Z*-score	0.37 (−0.33–1.14)	−0.06 (−0.72–0.67)	<0.01
Urine volume (mL/kg/24 h)	19.38 (11.76–23.81)	22.04 (15.69–30.00)	<0.01
Creatinine (mg/dL)	0.78 (0.65–0.85)	0.56 (0.45–0.67)	<0.01
Cholesterol (mg/dL)	155 (138–188)	171 (143–185.5)	NS
Glucose (mg/dL)	90 (83–93)	88 (84–93)	NS
BRI (L^−1^)	0.94 (0.08–4.16)	1.01 (0.16–3.89)	NS
Urine pH	6.28 (5.92–6.50)	6.34 (6.08–6.61)	NS
GFR (mL/min/1.73 m^2^)	122.32 (104.83–134.97)	125.42 (100.95–149.22)	NS
Oxalate/cr. (mmol/g cr.)	0.41 (0.17–0.60)	0.49 (0.26–0.73)	<0.01
Citrate/cr. (mg/g cr.)	291.94 (189.80–420.99)	502.75 (317.30–767.81)	<0.01
Ca/cr. (mmol/g cr.)	3.24 (1.45–5.38)	3.66 (2.20–5.92)	<0.05
P/cr. (mmol/g cr.)	16.51 (13.75–20.47)	20.77 (16.38–26.82)	<0.01
Mg/cr. (mmol/g cr.)	2.78 (2.11–3.45)	3.52 (2.67–4.62)	<0.01
Uric acid/cr.	0.36 (0.31–0.43)	0.46 (0.37–0.59)	<0.01
Ca/citrate	0.41 (0.20–0.78)	0.30 (0.16–0.56)	<0.05
Fractional excretion of uric acid (%)	4.02 (3.60–4.94)	6.08 (5.04–7.60)	<0.01

Data are presented as the median with the IQR in parenthesis, unless otherwise indicated

HU+, hyperuricemia; HU−, normouricemia

^a^The chi-square statistic was used

**Table 5:** Metabolic characteristics of reference patients with hyperuricemia and reference patients with normouricemia

Metabolic parameters	Reference group (HU+)	Reference group (HU−)	*p*
Oxalate/cr. (mmol/g cr.)	0.23 (0.15–0.30)	0.31 (0.23–0.39)	<0.01
Citrate/cr. (mg/g cr.)	501.53 (448.83–609.57)	654.69 (524.55–866.23)	<0.01
Ca/cr. (mmol/g cr.)	2.50 (1.86–3.00)	3.03 (2.30–3.80)	<0.01
P/cr. (mmol/g cr.)	17.22 (13.88–20.42)	21.61 (17.56–27.04)	<0.01
Mg/cr. (mmol/g cr.)	2.96 (2.51–3.42)	3.65 (3.10–4.31)	<0.01
Uric acid/cr.	0.37 (0.32–0.41)	0.45 (0.38–0.55)	<0.01
Ca/ citrate	0.19 (0.14–0.24)	0.17 (0.12–0.24)	NS

Data are presented as the median with the IQR in parenthesis, unless otherwise indicated

## Discussion

The present study was designed to determine the effect of obesity and metabolic disturbances on the urinary lithogenic risk profile in a large cohort of pediatric patients. In the first analysis, we confirmed that in comparison to the healthy children and adolescents of the reference group, the patients with urolithiasis had hypocitraturia, hypercalciuria and hyperoxaluria, as well as an increased BRI. This result is in agreement with published data. Many large epidemiologic studies have demonstrated that hypercalciuria is the most common metabolic abnormality in children with urolithiasis [31, 32]. Other studies have indicated that approximately 2–36 % of patients with pediatric urolithiasis have hyperoxaluria [33–35] and that there is a large prevalence of hypocitraturia in patients with urolithiasis, ranging from 8 to 68.3 % [36]. It is interesting to note that in our study we were able to demonstrate that serum cholesterol level in the urolithiasis group was significantly higher than that in the reference group. These findings are consistent with those of recent studies reporting associations between kidney stones and traditional atherosclerotic risk factors, including dyslipidemia and obesity [20, 37].

The second analysis was designed to test our hypothesis that children and adolescents with urinary stones and an increased BMI* Z*-score (overweight/obese) have different urinary risk lithogenic profiles than patients with urolithiasis and a normal body weight. However, contrary to expectations, we did not find a significant difference between these two groups.

Little is known about the biochemical mechanisms that would explain the association between obesity and urolithiasis. Recent studies have demonstrated that an increased body weight is related with changes in the biochemical components of urine, including phosphate, oxalate, uric acid and citrate [38–41]. Negri et al. [40] reported that uric acid and oxalate were significantly higher in the urine samples of obese patients, and Taylor et al. [42] showed a positive relationship between BMI and urinary excretion of oxalate, calcium, uric acid, citrate, sodium, phosphate and potassium. However these studies only demonstrated that obese patients are more predisposed to urolithiasis than those within the normal weight range. In our study we also confirmed that obese patients with urolithiasis had more hypocitraturia and hyperoxaluria than the reference group—although we found no such differences between the former group and urinary stone patients of normal weight. One possible explanation is that our patients were not extremely obese, with most being clinically overweight (*n* = 76) and only 21 patients being clinically obese. Powell et al. [41] demonstrated that patients with urolithiasis who weighed more than 120 kg excreted more oxalate, calcium and uric acid in than urine than patients weighing less than 100 kg. Similarly, Semins et al. [38] demonstrated that only significant obesity (BMI > 30 kg/m^2^) was associated with a significantly greater likelihood of being diagnosed with a kidney stone. In another study in adult stone formers Taylor et al. [42] found a positive association between BMI and urinary oxalate excretion in women, and with urinary calcium excretion in men.

It has been proposed that an increase in BMI causes a significant decrease in urinary pH level [42]. We confirmed this proposal in our study. Maalouf et al. [43] found that in urinary stone patients, urinary pH had a strong graded inverse association with BMI. In contrast, Nouvenne et al. [44] reported that among their study population there was no significant change in urinary pH with increasing BMI in both patients with urolithiasis and the healthy control group. The underlying factors explaining the fall in urine pH with increasing BMI in urolithiasis patients is not yet well explained. One possible explanation could be insulin resistance [45] as it has been proposed that hyperinsulinemia could lead to decreased urinary citrate level as well as increased levels of lithogenic factors in urine, including calcium, uric acid and oxalate [39, 45, 46]. In contrast, in their study, Dwyer et al. [47] did not observe significant trends in obesity in adolescent stone-formers. In a similar large-scale study, Penido et al. [48] also demonstrated that the incidence of obesity in pediatric patients with urolithiasis was not higher than that in the general population. Similar observations were reported by Kieran et al. [49] who described that an increased BMI did not correlate with earlier stone development, large stones or the need for multiple procedures in a stone-forming population.

We noted that our patients were indeed hyperuricemic, but that uric acid excretion did not differ between patients with urolithiasis and overweight or obesity and with urolithiasis and a normal body weight.

During our study we noticed that hyperuricemia—but not the BMI* Z*-score—influenced the urinary lithogenic risk profile in pediatric patients with urolithiasis. After taking serum uric acid levels into account, we extended our observation by showing that there was a significant decrease of crystallization inhibitors in hyperuricemic children and adolescents. It is interesting to note that we found a significant correlation between serum uric acid level and the urinary excretion of citrates and magnesium, which to our knowledge is the first observation of such a relationship.

The influence of dietary habits on urinary stone formation has been widely reported in the literature. It would appear that the consumption of animal protein predisposes to the development of hyperuricemia, thereby creating an acid load, which increases the urinary excretion of calcium and uric acid and reduced citrate [50].

A limitation of this study was the relatively small sample size of obese and overweight patients, when compared to those of normal weight. Similarly, there was a disproportion in the numbers of patients with hyperuricemia and those with normouricemia. Further work needs to be done in patients with hyperuricemia, but without urinary stones, to establish whether they are predisposed to urolithiasis and, if so, how it can be prevented.

In conclusion, hyperuricemia is associated with a decrease in the excretion of crystallization inhibitors in the urine. However, the clinical relevance of this observation needs to be confirmed in further studies. Among our patient cohort, obesity and overweight had no direct influence on the lithogenic risk profile in urinary stone formers, However it is possible that higher serum levels of uric acid were associated with impairment in renal function, which in turn could influence the excretion of lithogenic parameters.
